# Web-Based Psychoeducation Program for Improving Coping Mechanisms Among Family Caregivers of People with Schizophrenia: A Quasi-Experimental Study

**DOI:** 10.3390/healthcare14182888

**Published:** 2026-09-08

**Authors:** Ade Herman Surya Direja, Faridah Mohd Said, Jayasree S. Kanathasan, Tria Nopi Herdiani

**Affiliations:** 1 School of Nursing and Applied Science, Lincoln University College, Petaling Jaya 47301, Malaysia; faridah.msaid@lincoln.edu.my (F.M.S.); k.jayasree@lincoln.edu.my (J.S.K.); 2 Institute of Health Sciences (STIKES) Tri Mandiri Sakti, Bengkulu 38211, Indonesia; tria.phdscholar@lincoln.edu.my

**Keywords:** digital psychoeducation, schizophrenia, family caregivers, coping mechanisms, mental health nursing, digital health, caregiver support

## Abstract

Background/Objectives: Family caregivers of people with schizophrenia experience substantial psychological and practical demands associated with long-term caregiving. This study examined whether participation in a web-based digital psychoeducation program was associated with longitudinal changes in coping mechanisms among family caregivers of people with schizophrenia. Methods: A non-randomized quasi-experimental pretest–posttest control-group study was conducted at a mental health hospital in Indonesia. The final analytic sample comprised 202 family caregivers of people with schizophrenia, with 101 participants in the intervention group and 101 in the control group. The intervention comprised 10 structured web-based psychoeducation modules delivered over 10 weeks. Coping was assessed using the Brief COPE Inventory and summarized into problem-focused, emotion-focused, and avoidance coping domains. Linear mixed-effects models were used as the primary adjusted longitudinal analysis, with study group, time, group-by-time interaction, age group, and marital status included as fixed effects. Wilcoxon signed-rank and Mann–Whitney U tests were retained as complementary unadjusted analyses, while baseline-adjusted linear regression models were used as sensitivity analyses. Results: Significant group-by-time interactions were observed for problem-focused coping and emotion-focused coping. The adjusted between-group difference in change was 0.450 points (95% CI: 0.357–0.544; partial ηp^2^ = 0.312; *p* < 0.001) for problem-focused coping and 0.263 points (95% CI: 0.137–0.390; partial ηp^2^ = 0.078; *p* < 0.001) for emotion-focused coping. In contrast, no statistically significant differential change was observed for avoidance coping (adjusted difference in change = 0.074 points, 95% CI: −0.055 to 0.204; partial ηp^2^ = 0.006; *p* = 0.259). Baseline-adjusted sensitivity analyses produced a consistent overall pattern. Conclusions: Participation in the 10-week web-based digital psychoeducation program was associated with greater longitudinal improvements in problem-focused and emotion-focused coping, but not avoidance coping. Given the non-randomized design and baseline differences between groups, these findings should be interpreted as adjusted longitudinal associations rather than definitive evidence of causal efficacy. The program may therefore represent an accessible complementary approach to supporting caregiver adaptation in long-term schizophrenia care.

## 1. Introduction

Schizophrenia is a severe and chronic psychiatric disorder characterized by disturbances in perception, cognition, emotion, and behavior, often manifested through hallucinations, delusions, disorganized thinking, and impaired social functioning [1,2]. Affecting approximately 24 million people worldwide, schizophrenia represents a substantial public health challenge due to its long-term treatment requirements and need for continuous psychosocial support [3]. Although pharmacological treatment remains the cornerstone of schizophrenia management, long-term care and functional support often require active involvement from family caregivers, particularly in low- and middle-income countries where community mental health resources remain limited [4].

Family caregivers play an essential role in supporting individuals with schizophrenia by assisting with medication adherence, monitoring symptoms, providing emotional support, and managing daily activities [5]. However, these responsibilities frequently expose caregivers to considerable psychological distress, emotional exhaustion, social isolation, financial burden, and reduced quality of life [5,6,7]. Caregivers of people with schizophrenia often experience substantial caregiving burden due to symptom unpredictability, recurrent relapses, behavioral disturbances, and persistent societal stigma [8]. Consequently, interventions that support caregivers’ psychological well-being have become an important component of comprehensive schizophrenia care [9].

Coping mechanisms represent an important factor influencing how caregivers manage caregiving-related stress and maintain psychological well-being [10,11]. Coping mechanisms refer to cognitive and behavioral strategies used by individuals to manage internal and external demands arising from stressful situations [12]. In the context of schizophrenia caregiving, coping responses may involve problem-focused coping, emotion-focused coping, and avoidance coping, reflecting different ways caregivers respond to caregiving-related stress. Less adaptive coping responses, including excessive avoidance, denial, and emotional withdrawal, may increase psychological distress and interfere with effective caregiving [13,14]. Therefore, strengthening adaptive coping mechanisms among family caregivers represents a key objective of psychosocial interventions.

Coping mechanisms are also closely related to broader dimensions of the caregiving experience, including perceived stress, caregiver burden, resilience, psychological well-being, and quality of life [6,15,16]. Problem-focused strategies may facilitate active management of caregiving demands, whereas emotion-focused strategies may support the regulation of emotional responses when stressors cannot be readily modified [10,17]. In contrast, persistent reliance on avoidance-oriented strategies may be associated with less adaptive responses to prolonged caregiving demands [18,19]. These relationships highlight the importance of examining coping not only as an individual behavioral response but also as a component of broader caregiver adaptation and well-being. Caregivers’ capacity to manage persistent caregiving demands may also have implications for their sustained involvement in supportive and person-centered care, although caregiver coping and quality of care represent distinct constructs that require separate assessment.

Family psychoeducation has consistently been recommended as an evidence-based intervention for schizophrenia care. Psychoeducation improves caregivers’ understanding of schizophrenia, enhances communication and problem-solving skills, strengthens emotional regulation, and promotes collaborative management between families and healthcare professionals [20,21,22]. Previous studies have demonstrated that psychoeducation can reduce caregiver burden, improve illness-related knowledge, enhance caregiving skills, and strengthen family involvement in schizophrenia care [23]. Nevertheless, conventional face-to-face psychoeducation programs often encounter substantial barriers, including geographical limitations, transportation difficulties, scheduling conflicts, workforce shortages, and limited continuity of caregiver participation.

The rapid expansion of digital health technologies has created new opportunities to deliver psychoeducation through web-based platforms. Web-based psychoeducation may extend access to structured educational resources while providing opportunities for caregivers to acquire knowledge, reflect on caregiving experiences, and develop coping strategies [24,25]. Through flexible and self-directed learning, web-based interventions may help caregivers acquire and apply coping strategies within their daily caregiving experiences. These characteristics make digital psychoeducation particularly valuable for caregivers who face geographical, occupational, or logistical barriers to attending conventional psychoeducation sessions [26].

Despite the increasing use of digital approaches in mental health care, evidence regarding the association between web-based digital psychoeducation and coping mechanisms among family caregivers of people with schizophrenia remains limited. Previous studies have primarily focused on broader caregiver outcomes, including caregiver burden, psychological distress, and illness-related knowledge, while fewer studies have examined changes in specific coping mechanisms following structured web-based psychoeducational interventions [27]. Although caregiver burden represents an important indicator of the psychological impact of caregiving, coping mechanisms reflect the underlying processes through which caregivers perceive, regulate, and respond to ongoing caregiving demands. Adaptive coping strategies are closely related to how caregivers manage stressors and maintain psychological well-being while providing continuous support for individuals with schizophrenia. Therefore, examining specific coping domains may provide a more focused understanding of caregiver adaptation in the context of psychoeducational support. Evidence from low- and middle-income settings, particularly Indonesia, remains limited [28], despite the growing need for accessible caregiver support strategies that can overcome barriers related to time, distance, and availability of mental health services [29].

The multidimensional nature of caregiver adaptation also underscores the importance of selecting outcome measures with psychometric properties appropriate to the construct being examined. Instruments such as the Zarit Burden Interview, which assesses caregiver burden, and the CarerQol, which assesses care-related quality of life, illustrate the use of construct-specific measures for evaluating different dimensions of the caregiving experience [30,31]. Psychometric evidence for caregiver-reported measures may vary across instrument versions and populations, reinforcing the importance of considering reliability and validity in the context in which an instrument is applied. In the present study, coping was the predefined outcome of interest and was therefore assessed using the Brief COPE Inventory; caregiver burden and care-related quality of life were not measured as study outcomes.

This study aimed to examine changes in coping mechanisms associated with participation in a web-based digital psychoeducation program among family caregivers of people with schizophrenia using a non-randomized quasi-experimental design. The intervention was developed as a structured web-based psychoeducation program providing evidence-based information and practical strategies related to schizophrenia, caregiving skills, stress management, communication, relapse prevention, resilience, and emotional well-being. The study examined changes in problem-focused coping, emotion-focused coping, and avoidance coping from pre-intervention to post-intervention and compared these changes between the intervention and control groups. Although caregiver burden, psychological distress, quality of life, resilience, perceived stress, social support, and caregiving competence represent important dimensions of caregiver well-being, the present study specifically focused on coping mechanisms as the predefined primary outcome because coping reflects the cognitive and behavioral processes through which caregivers respond to ongoing caregiving demands. Given the non-randomized design and observed baseline differences between the study groups, the analytical strategy incorporated longitudinal adjustment for relevant sociodemographic covariates, with additional baseline-adjusted analyses used to examine the robustness of the findings. The findings are intended to contribute to the limited evidence on longitudinal changes in specific coping domains associated with participation in web-based digital psychoeducation among family caregivers involved in long-term schizophrenia care.

## 2. Materials and Methods

### 2.1. Study Design

This study employed a non-randomized quasi-experimental pretest–posttest control-group design to examine changes in coping mechanisms among family caregivers of people with schizophrenia. The study included an intervention group and a concurrent control group, with coping outcomes assessed at two time points: before the intervention (pre-intervention) and after completion of the 10-week study period (post-intervention).

Participants in the intervention group received a 10-week web-based digital psychoeducation program delivered through the Ademind platform, whereas participants in the control group did not receive the study-related digital psychoeducation program during the study period. The intervention comprised 10 structured psychoeducational modules designed to provide caregivers with knowledge and practical strategies related to schizophrenia caregiving, stress management, communication, relapse prevention, resilience, and emotional well-being. Changes in problem-focused coping, emotion-focused coping, and avoidance coping were evaluated across the two assessment time points.

Participants were not randomly allocated to the intervention or control group. Group allocation was based on participants’ willingness to participate in the 10-week web-based psychoeducation program. Eligible caregivers who agreed to undertake the digital psychoeducation program were assigned to the intervention group, whereas those who declined the intervention but agreed to participate in the study assessments were assigned to the control group. Caregivers who declined both the intervention and participation in the study were not enrolled. Recruitment continued until the predetermined sample size of 101 participants per group was achieved.

Given the non-randomized allocation procedure, equivalence between the intervention and control groups at baseline could not be assumed. Baseline sociodemographic characteristics and coping outcomes were therefore compared between groups to identify potential pre-intervention differences. To account for the repeated-measures structure of the data and potential confounding associated with observed baseline sociodemographic differences, linear mixed-effects models were used as the primary adjusted longitudinal analysis. Separate models were fitted for problem-focused coping, emotion-focused coping, and avoidance coping, with study group, assessment time, and the group-by-time interaction included as fixed effects and age group and marital status included as covariates. Repeated measurements were modeled within participants to account for the correlation between pre- and post-intervention observations.

The group-by-time interaction represented the primary effect of interest because it directly evaluated whether the magnitude of change in coping outcomes over time differed between the intervention and control groups after adjustment for age group and marital status. This analytical approach was selected to provide a more appropriate assessment of differential longitudinal change than separate within-group pre–post comparisons alone, particularly given the non-randomized design and observed baseline differences between groups. Additional baseline-adjusted linear regression models were conducted as sensitivity analyses to examine the robustness of the primary findings using an alternative adjustment strategy.

The non-randomized quasi-experimental design was selected to evaluate changes in caregivers’ coping mechanisms under real-world mental healthcare conditions while maintaining a concurrent comparison group. However, because treatment allocation was not randomized and residual confounding cannot be completely excluded through statistical adjustment, the findings were interpreted as longitudinal changes associated with participation in the web-based psychoeducation program rather than as definitive evidence of causal efficacy.

### 2.2. Study Setting

The study was conducted at Soeprapto Mental Hospital, Bengkulu, Indonesia. The hospital provides specialized mental health services for people with schizophrenia and other psychiatric conditions and serves as an important mental healthcare facility in Bengkulu Province.

The hospital was selected as the study setting because it provides psychiatric services for people with schizophrenia and serves as an access point for identifying eligible family caregivers involved in patient care. Participant recruitment and baseline and post-intervention assessments were conducted through the hospital setting, while the digital psychoeducation intervention was delivered remotely through the Ademind.online web-based platform.

### 2.3. Data Collection Procedure

Eligible caregivers were informed about the study objectives, procedures, potential benefits, and their rights as research participants. After providing written informed consent, participants were enrolled in the study and completed the baseline assessment (pretest), which included demographic characteristics and assessment of coping mechanisms.

Following the pretest, participants in the intervention group received an orientation on how to access and use the Ademind.online web-based platform. The orientation was provided to ensure that participants could navigate the platform and access the digital psychoeducation materials appropriately.

The intervention group subsequently completed the ten digital psychoeducation modules over a 10-week intervention period. Each module required approximately 30 min to complete, with one module assigned for completion each week. Participant engagement was monitored using a program participation monitoring sheet to document module completion and participation throughout the intervention period.

The control group did not receive access to the web-based digital psychoeducation intervention or any study-related psychoeducational intervention during the study period. Following completion of the 10-week intervention period, participants in both groups completed the posttest using the same coping assessment instrument administered at baseline. The pretest and posttest data were subsequently analyzed to assess changes in caregivers’ coping mechanisms.

### 2.4. Participants, Sample Size, and Sampling

The target population comprised family caregivers of people with schizophrenia receiving treatment at Soeprapto Mental Hospital, Bengkulu, Indonesia. According to hospital records, the recruitment frame consisted of 1208 potentially eligible family caregivers. This population size was used solely to define the recruitment frame and was not used to determine the analytical sample size for the intervention study.

Sample size adequacy was evaluated using G*Power version 3.1.9.7 (Heinrich Heine University Düsseldorf, Düsseldorf, Germany). A sensitivity power analysis was conducted based on the achieved sample of 202 participants for a repeated-measures analysis with a within–between interaction, corresponding to two study groups (intervention and control) assessed at two time points (pre- and post-intervention). The analysis assumed a significance level of α = 0.05, statistical power of 80%, a correlation of 0.50 between repeated measurements, and a nonsphericity correction of ε = 1.00. Under these assumptions, the achieved sample of 202 participants (101 per group) corresponded to an approximate minimum detectable interaction effect of Cohen’s *f* = 0.099.

Because this calculation was performed as a sensitivity analysis based on the achieved sample rather than as an a priori sample size calculation, no anticipated dropout rate was incorporated into the power analysis. Accordingly, Cohen’s *f* = 0.099 represents the approximate minimum detectable interaction effect under the specified assumptions. The recruitment frame of 1208 caregivers and a margin-of-error approach were not used to establish the analytical sample size.

Participants were recruited using a non-probability quota sampling technique. Equal recruitment quotas were used for the intervention and control groups, resulting in a final analytic sample of 101 participants with complete study assessments in each group. Because random allocation was not feasible within the routine clinical setting, group assignment followed the non-randomized allocation procedure described in Section 2.1. Caregivers who agreed to participate in the 10-week web-based digital psychoeducation program were assigned to the intervention group, whereas those who declined the intervention but agreed to participate in the study assessments were assigned to the control group.

Participants were eligible if they were family caregivers of individuals with schizophrenia who had received treatment at Soeprapto Mental Hospital for at least five years. This criterion referred to the treatment history of the individual with schizophrenia rather than the duration of caregiving. Eligible caregivers were required to be actively involved in caring for their family member, willing to participate and provide written informed consent, able to access an internet-connected digital device, and able to complete the study procedures. Participants were excluded if they were not actively involved in caregiving activities, were unable to complete the study procedures, or lacked access to the digital device or internet connection required to access the web-based psychoeducation program.

**Figure 1:**
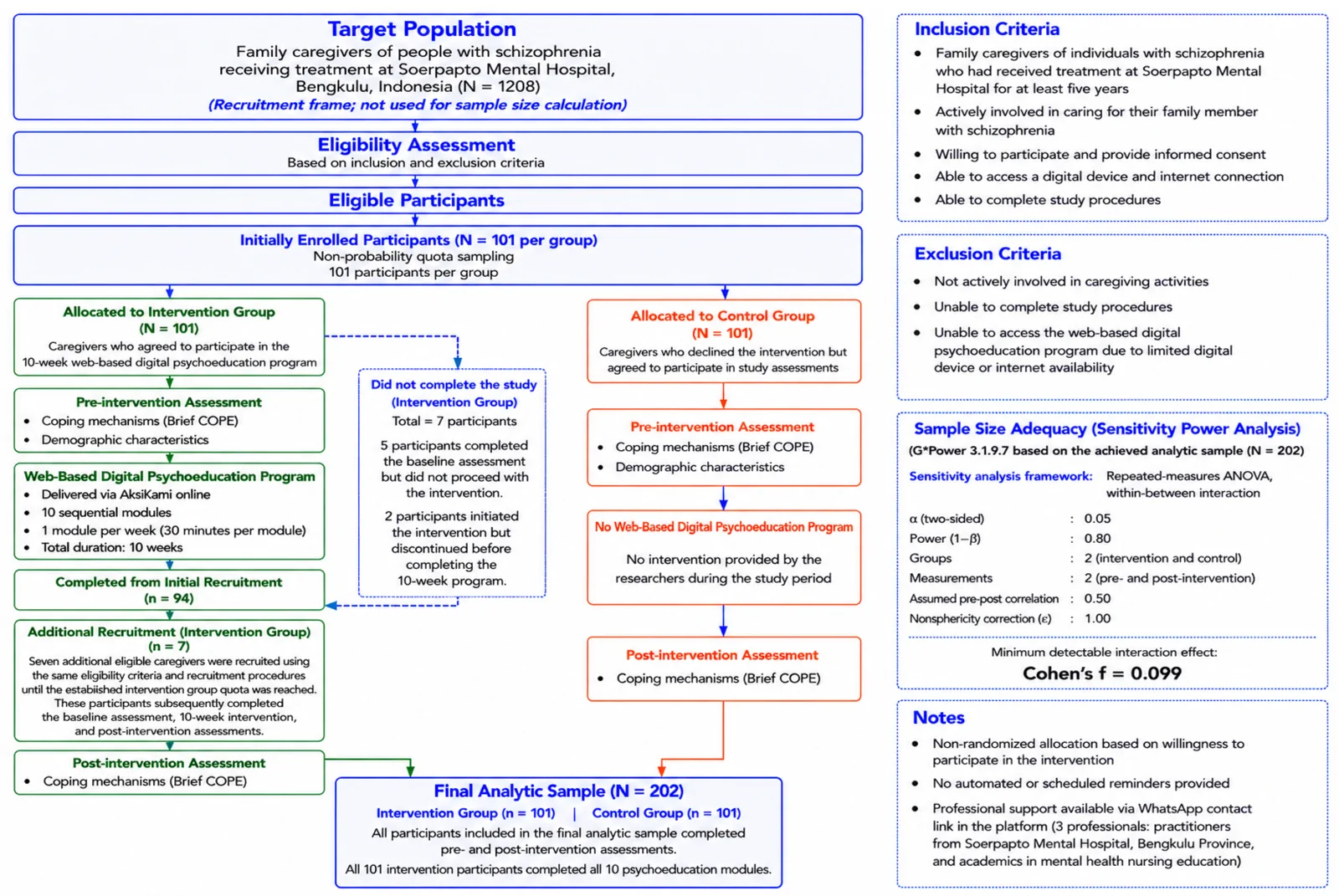
Participant recruitment, group allocation, intervention, and assessment flow.

The intervention group initially comprised 101 participants. During recruitment and intervention delivery, seven of these participants did not complete the study protocol (6.9%). Five participants completed the baseline assessment but did not proceed with the intervention, while two participants initiated the intervention but discontinued before completing the 10-week program. None of these seven participants completed the post-intervention assessment. To maintain the established recruitment quota of 101 participants in the intervention group, seven additional eligible caregivers were subsequently recruited using the same eligibility criteria and recruitment procedures. These additional participants completed the study protocol, resulting in a final analytic sample of 101 participants in the intervention group with complete pre- and post-intervention assessments. The participant recruitment, group allocation, intervention, and assessment procedures are summarized in Figure 1.

### 2.5. Web-Based Digital Psychoeducation Intervention

The intervention consisted of a structured web-based digital psychoeducation program developed specifically for family caregivers of people with schizophrenia. The program was delivered through the Ademind.online platform and was designed to provide caregivers with accessible and structured learning materials addressing schizophrenia, caregiving practices, stress management, social support, communication, relapse prevention, long-term care planning, resilience, and emotional well-being.

The psychoeducation program consisted of ten sequential modules. The content was organized to progressively improve caregivers’ understanding of schizophrenia and develop practical and psychological skills relevant to long-term caregiving. Each module contained specific learning materials and learning outcomes designed to facilitate knowledge acquisition, reflection, and the application of caregiving strategies in daily situations. The structure, key content, and expected learning outcomes of the ten modules are presented in Table 1.

Each module was designed to require approximately 30 min to complete, and participants were given one week to complete each module. The intervention was delivered asynchronously through the Ademind.online platform, allowing caregivers to access learning materials using an internet-connected digital device according to their individual circumstances. The intervention period consisted of ten weeks, with one module made available for completion each week.

Each module combined educational materials with video content and structured activities relevant to its learning objectives. Participants were not limited to reviewing written information but were also guided through practical and reflective exercises intended to relate the material to situations encountered in caregiving. These activities included relaxation and deep-breathing exercises, communication simulations, reflection on caregiving experiences, and care-planning activities. For example, the stress-management module incorporated mindfulness and breathing exercises, whereas the communication module included simulated practice in active listening, validation, and non-verbal communication. Learning activities were tailored to the objectives of individual modules rather than applied uniformly across all sessions. End-of-module questions were used to assess participants’ understanding and encourage reflection on the application of the material to their caregiving experiences.

Adherence to the intervention was monitored throughout the 10-week program using records from the web-based platform and participation monitoring sheets. Completion was assessed at the module level. A module was considered completed when the participant had accessed the full module content, reviewed the educational materials, viewed the available video content, completed the designated exercises or activities, and submitted the end-of-module evaluation questions. These criteria were applied throughout the intervention period to document participants’ progression across the ten modules. During the intervention period, participants could contact three designated professionals through a WhatsApp contact link integrated into the web-based platform when they required clarification regarding the module content or its application to caregiving situations. The professional support team included practitioners from Soeprapto Psychiatric Hospital, Bengkulu Province, and academics with expertise in mental health nursing education. Contact was available on an as-needed basis and was intended to provide clarification and supportive guidance related to the psychoeducation program rather than a separate therapeutic or clinical intervention. No automated or scheduled reminders were provided to prompt participants to access or complete the weekly modules.

The development of the web-based digital psychoeducation program followed the ADDIE (Analysis, Design, Development, Implementation, and Evaluation) framework [32]. The intervention content was developed based on the identified needs of family caregivers of people with schizophrenia and was reviewed by a multidisciplinary expert panel consisting of a psychiatrist, a clinical psychologist, a senior mental health nurse, and an academic specialist in psychiatric nursing. Content validity was assessed using the Content Validity Index (CVI). The S-CVI/Ave value was 0.96, indicating a high level of content validity. Revisions were made based on expert feedback to improve the clarity, relevance, and contextual appropriateness of the learning materials.

The digital platform was evaluated by information technology specialists to assess its functionality, interface design, and technical reliability before implementation. A preliminary needs assessment involving 30 caregivers was conducted to evaluate the feasibility and acceptance of the web-based platform. Feedback obtained from the development and evaluation stages was used to refine the content structure and presentation of the psychoeducation modules before implementation in the main study.

The intervention was designed to strengthen caregivers’ coping mechanisms through knowledge acquisition, practical caregiving skills, stress management, communication strategies, social support, relapse prevention, long-term care planning, and resilience-building. Although avoidance coping was assessed as one of the study outcomes, it was not established as a specific intervention target. The three coping domains were assessed before and after the intervention to examine changes in caregivers’ coping mechanisms associated with participation in the web-based digital psychoeducation program.

### 2.6. Outcome Measures

The primary outcome of this study was caregivers’ coping mechanisms, assessed using the Brief COPE Inventory developed by Carver [33]. The Brief COPE is a 28-item self-report instrument comprising 14 two-item subscales: active coping, planning, use of instrumental support, use of emotional support, positive reframing, acceptance, humor, religion, self-distraction, denial, venting, behavioral disengagement, substance use, and self-blame [33,34]. Each item was rated on a four-point response scale ranging from 1 to 4, with higher scores indicating greater use of the corresponding coping strategy.

For the primary domain-level analyses, the 14 Brief COPE subscales were organized into three broader coping domains using a theory-informed framework adapted from Cooper et al. [35] and informed by the Indonesian Brief COPE validation literature [36]: problem-focused coping, emotion-focused coping, and an avoidance-oriented coping composite. Problem-focused coping comprised active coping, planning, and instrumental support; emotion-focused coping comprised emotional support, positive reframing, acceptance, religion, and humor; and the avoidance-oriented composite comprised self-distraction, denial, venting, behavioral disengagement, substance use, and self-blame. Cooper et al. originally described the third composite as dysfunctional coping; the term “avoidance-oriented coping” was used in the present study to reflect the conceptual emphasis on avoidant and maladaptive responses while retaining the same subscale composition.

The classification of self-blame within the avoidance-oriented coping composite domain was based on its conceptualization as a self-directed negative response to stressful circumstances rather than an adaptive strategy for regulating emotional distress or directly addressing the stressor. Similarly, venting was classified within the dysfunctional domain in accordance with the adopted classification framework. This distinction separates these responses from potentially adaptive emotion-focused strategies such as acceptance, positive reframing, emotional support, religion, and humor.

Importantly, the three-domain structure has not, to our knowledge, been specifically validated among Indonesian family caregivers of people with schizophrenia. An Indonesian validation study conducted among patients with advanced cancer evaluated the Cooper et al. three-domain model but did not demonstrate adequate model fit [36]. Therefore, the three-domain classification in the present study was treated as a theory-informed composite scoring framework rather than as a psychometrically confirmed factor structure for the study population.

Domain scores were calculated by averaging the item scores corresponding to the subscales assigned to each domain. Accordingly, problem-focused coping comprised six items, emotion-focused coping comprised 10 items, and the avoidance-oriented coping composite comprised 12 items. The use of mean scores retained the original 1–4 response metric and facilitated comparison across domains containing different numbers of items. Higher scores represented greater use of the coping strategies included within the respective domain. For brevity and readability, the avoidance-oriented coping composite is referred to as “avoidance coping” in the Results, Discussion, tables, and figures.

Internal consistency of the three composite domains was evaluated using baseline responses from the full study sample *n* = 202). Problem-focused coping demonstrated acceptable internal consistency (Cronbach’s α = 0.716), whereas emotion-focused coping showed lower internal consistency (α = 0.573), and the avoidance-oriented coping composite demonstrated poor internal consistency (α = 0.245). These coefficients were interpreted as sample-specific estimates of internal consistency rather than evidence supporting the structural validity of the three-domain model. Given the limited internal consistency of the emotion-focused composite and particularly the poor internal consistency of the avoidance-oriented coping composite, findings for these broader domains were interpreted cautiously and complemented by analyses of the original 14 Brief COPE subscales.

To complement the domain-level analyses and reduce reliance on the broader composite scores, all 14 original Brief COPE subscales were additionally examined separately as supplementary analyses. These subscale-level analyses were intended to provide a more granular assessment of strategy-specific coping responses and are reported in Supplementary Table S1.

The Brief COPE was administered at both pre-intervention and post-intervention assessments to evaluate changes in caregivers’ coping mechanisms over the study period. Demographic characteristics, including age, gender, marital status, educational level, employment status, and monthly family income, were also collected to characterize the study population. Age group and marital status were additionally considered in the adjusted analyses because significant between-group differences in these characteristics were identified at baseline.

### 2.7. Statistical Analysis

Data were analyzed using statistical procedures appropriate to the non-randomized pretest–posttest design and the distributional characteristics of the study variables. Descriptive statistics were used to summarize participant characteristics and coping outcomes. Categorical variables were presented as frequencies and percentages, whereas continuous variables were summarized using means and standard deviations (SDs) and, where appropriate, medians and interquartile ranges (IQRs). Baseline sociodemographic characteristics and coping scores were compared between the intervention and control groups to assess pre-intervention between-group differences. Categorical variables were compared using the chi-square test or an appropriate alternative when expected cell counts were insufficient. Baseline coping outcomes were compared using the Mann–Whitney U test because their distributions did not consistently satisfy the assumptions required for parametric comparisons.

For complementary unadjusted analyses, within-group changes in problem-focused coping, emotion-focused coping, and avoidance coping from pre-intervention to post-intervention were evaluated using the Wilcoxon signed-rank test. Change scores (Δ) were calculated as post-intervention minus pre-intervention scores, and unadjusted between-group differences in change were examined using the Mann–Whitney U test. Effect sizes for the Wilcoxon signed-rank and Mann–Whitney U tests were calculated as *r* = |Z|/√N, using the number of participants contributing to the corresponding comparison. These non-parametric analyses were retained to characterize within-group changes and unadjusted between-group differences but were not considered the principal analyses of intervention-associated longitudinal change.

Given the non-randomized group allocation, observed baseline imbalance, and repeated-measures structure of the data, linear mixed-effects models were used as the primary adjusted longitudinal analyses. Separate models were fitted for problem-focused coping, emotion-focused coping, and avoidance coping. Each model included study group (intervention vs. control), assessment time (pre-intervention vs. post-intervention), and the group-by-time interaction as fixed effects. Age group and marital status were included as covariates because significant between-group differences in these characteristics were identified at baseline. Repeated observations were modeled within participants to account for the correlation between pre- and post-intervention measurements from the same participant.

The group-by-time interaction was specified as the primary effect of interest because it directly tested whether longitudinal changes in each coping outcome differed between the intervention and control groups after adjustment for age group and marital status. Adjusted estimated marginal means (EMMs), standard errors (SEs), and 95% confidence intervals (CIs) were obtained for each group at each assessment time. Type III tests of fixed effects were used to evaluate group, time, and group-by-time effects, with F statistics, denominator degrees of freedom, and corresponding *p*-values reported. The adjusted between-group difference in change was estimated from the group-by-time interaction contrast and reported with its 95% CI to quantify the magnitude and precision of the differential longitudinal change.

Effect sizes for the group-by-time interactions were quantified using partial eta squared (ηp^2^), derived from the corresponding F statistic and degrees of freedom. For descriptive interpretation, ηp^2^ values of approximately 0.01, 0.06, and 0.14 were considered indicative of small, medium, and large effects, respectively. Effect sizes were interpreted together with the adjusted estimates, 95% CIs, and *p*-values rather than as isolated thresholds.

As a sensitivity analysis, separate baseline-adjusted linear regression models were fitted for each post-intervention coping outcome. Each model included study group, the corresponding baseline coping score, age group, and marital status as predictors. Unstandardized regression coefficients (B) with 95% CIs were reported. These models provided an alternative baseline-adjustment strategy and were used to assess whether the pattern of findings was consistent with that obtained from the primary linear mixed-effects models.

Given concerns regarding the internal consistency of the broader Brief COPE composite domains, all 14 original Brief COPE subscales were additionally examined to provide a more granular assessment of strategy-specific coping responses. Pre-to-post change scores were calculated for each subscale, and between-group differences in these change scores were assessed using the Mann–Whitney U test. Because the 14 subscale-level comparisons constituted multiple supplementary tests, Holm’s sequential procedure was applied to control the family-wise error rate. Effect sizes for these comparisons were calculated as *r* = |Z|/√N. Both unadjusted and Holm-adjusted *p*-values were reported, with findings interpreted primarily on the basis of the multiplicity-adjusted results.

Missing data were assessed at the item level for all 28 Brief COPE items at both pre-intervention and post-intervention assessments. All 202 participants included in the final analytic sample had complete Brief COPE data at both assessment time points. The seven initially recruited intervention participants who did not complete the study protocol and did not provide post-intervention data were not included in the final analytic sample. Accordingly, the primary adjusted longitudinal analyses, sensitivity analyses, and complementary analyses were conducted using complete pre- and post-intervention data from the final analytic sample (*n* = 202). No additional imputation procedures were therefore applied to the analyzed dataset.

All statistical tests were two-tailed, and a *p*-value < 0.05 was considered statistically significant for the primary analyses. For the supplementary 14-subscale analyses, statistical significance was evaluated after Holm adjustment as described above. Statistical analyses were conducted using IBM SPSS Statistics version 26.0 (IBM Corp., Armonk, NY, USA).

### 2.8. Ethical Considerations

Ethical approval for this study was obtained from the Research Ethics Committee of STIKES Tri Mandiri Sakti Bengkulu and KEPPIN under Research Ethics Approval No. 000197/KEPK STIKES TMS BENGKULU/2025. Before enrollment, all participants were informed about the purpose, procedures, potential benefits, and voluntary nature of the study and provided written informed consent.

Participation was voluntary, and participants were informed of their right to withdraw from the study at any stage without consequences. Confidentiality and anonymity were maintained throughout the research process. All information obtained from participants was treated as confidential and used solely for research purposes. The study procedures were conducted in accordance with applicable ethical principles for research involving human participants.

## 3. Results

### 3.1. Participant Characteristics and Study Completion

A total of 202 family caregivers were included in the final analytic sample, comprising 101 participants in the intervention group and 101 participants in the control group. Participants were characterized according to gender, age, marital status, educational level, occupation, and monthly family income. These characteristics were assessed to describe the study sample and examine baseline differences between the intervention and control groups. The baseline sociodemographic characteristics of participants are presented in Table 2.

Significant differences were observed between the intervention and control groups in age group (χ^2^ = 23.214, *p* < 0.001) and marital status (χ^2^ = 6.996, *p* = 0.008). The intervention group included a greater proportion of younger caregivers, whereas the control group included a greater proportion of caregivers in older age categories. The proportion of participants classified as not married was also higher in the intervention group than in the control group. No statistically significant between-group differences were observed for sex, educational level, occupation, or monthly family income.

The intervention group initially comprised 101 participants. Seven of these participants (6.9%) did not complete the study protocol and were not included in the final analysis. Five participants completed the baseline assessment but did not proceed with the intervention, while two participants initiated the intervention but discontinued before completing the 10-week program. To maintain the established intervention-group quota, seven additional eligible caregivers were subsequently recruited using the same eligibility criteria and recruitment procedures. The final analytic sample therefore comprised 101 participants in the intervention group who completed the 10-week intervention and post-intervention assessment. Among these 101 participants, all completed all ten web-based psychoeducation modules, corresponding to a 100% module-completion rate among participants included in the final analytic sample. Accordingly, the completion rate for each of the ten modules was 100% among these participants. Module completion was verified using platform records and participation monitoring sheets.

### 3.2. Baseline Coping Outcomes

Table 3 presents the baseline coping scores for the intervention and control groups. Problem-focused coping differed significantly between groups at baseline. The intervention group had a mean score of 2.67 (SD = 0.55) and a median (IQR) of 2.67 (0.67), compared with a mean score of 2.52 (SD = 0.21) and a median (IQR) of 2.50 (0.33) in the control group (U = 3493.50, *p* < 0.001).

Emotion-focused coping did not differ significantly between groups at baseline. The intervention group had a mean score of 2.41 (SD = 0.47) and a median (IQR) of 2.50 (0.70), whereas the control group had a mean score of 2.39 (SD = 0.49) and a median (IQR) of 2.40 (0.75) (U = 4853.50, *p* = 0.551).

No significant baseline difference was observed for avoidance coping. The intervention group had a mean score of 2.14 (SD = 0.36) and a median (IQR) of 2.17 (0.58), compared with a mean score of 2.18 (SD = 0.35) and a median (IQR) of 2.17 (0.50) in the control group (U = 4859.00, *p* = 0.560).

The baseline assessment therefore identified a significant between-group difference in problem-focused coping, while emotion-focused and avoidance coping were comparable between groups. Given the non-randomized allocation, the baseline imbalance in problem-focused coping, together with the observed demographic differences, was accounted for in the adjusted longitudinal analyses presented in Section 3.3.

### 3.3. Primary Adjusted Longitudinal Analysis Using Linear Mixed-Effects Models

Linear mixed-effects models were used as the primary adjusted longitudinal analyses to examine whether changes in coping outcomes over time differed between the intervention and control groups. Separate models were fitted for problem-focused coping, emotion-focused coping, and avoidance coping, with study group, assessment time, and the group-by-time interaction included as fixed effects and age group and marital status included as covariates. The group-by-time interaction was specified as the primary effect of interest. Adjusted estimated marginal means (EMMs), between-group differences in change, 95% confidence intervals (CIs), and interaction effect sizes are presented in Table 4.

A statistically significant group-by-time interaction was observed for problem-focused coping, *F*(1, 200) = 90.878, *p* < 0.001, partial ηp^2^ = 0.312. The adjusted EMM increased from 2.577 at pre-intervention to 2.998 at post-intervention in the intervention group, whereas it changed from 2.381 to 2.351 in the control group. The adjusted between-group difference in change was 0.450 points (95% CI: 0.357–0.544), indicating a greater adjusted longitudinal increase in problem-focused coping in the intervention group relative to the control group.

A statistically significant group-by-time interaction was also observed for emotion-focused coping, *F*(1, 200) = 16.907, *p* < 0.001, partial ηp^2^ = 0.078. The adjusted EMM increased from 2.340 to 2.675 in the intervention group and from 2.336 to 2.407 in the control group. The adjusted between-group difference in change was 0.263 points (95% CI: 0.137–0.390), indicating a greater adjusted longitudinal increase in emotion-focused coping over time in the intervention group.

For avoidance coping, the group-by-time interaction was not statistically significant, *F*(1, 200) = 1.283, *p* = 0.259, partial ηp^2^ = 0.006. The adjusted EMM changed from 2.050 to 2.113 in the intervention group and from 2.078 to 2.066 in the control group. The adjusted between-group difference in change was 0.074 points (95% CI: −0.055 to 0.204). The confidence interval included zero, providing no evidence of a differential change in avoidance coping between groups after adjustment for age group and marital status.

The magnitude of the adjusted effects provided additional context for interpreting the statistical findings. The magnitude of the group-by-time interaction was large for problem-focused coping (partial ηp^2^ = 0.312) and medium for emotion-focused coping (partial ηp^2^ = 0.078). The corresponding adjusted between-group differences in change were 0.450 points (95% CI: 0.357–0.544) and 0.263 points (95% CI: 0.137–0.390), respectively. In contrast, the magnitude of the group-by-time interaction for avoidance coping was small (partial ηp^2^ = 0.006), and the adjusted difference in change was imprecisely estimated around zero (0.074 points, 95% CI: −0.055 to 0.204). These estimates provide information on the magnitude and precision of the observed group differences in longitudinal change beyond statistical significance alone.

**Figure 2:**
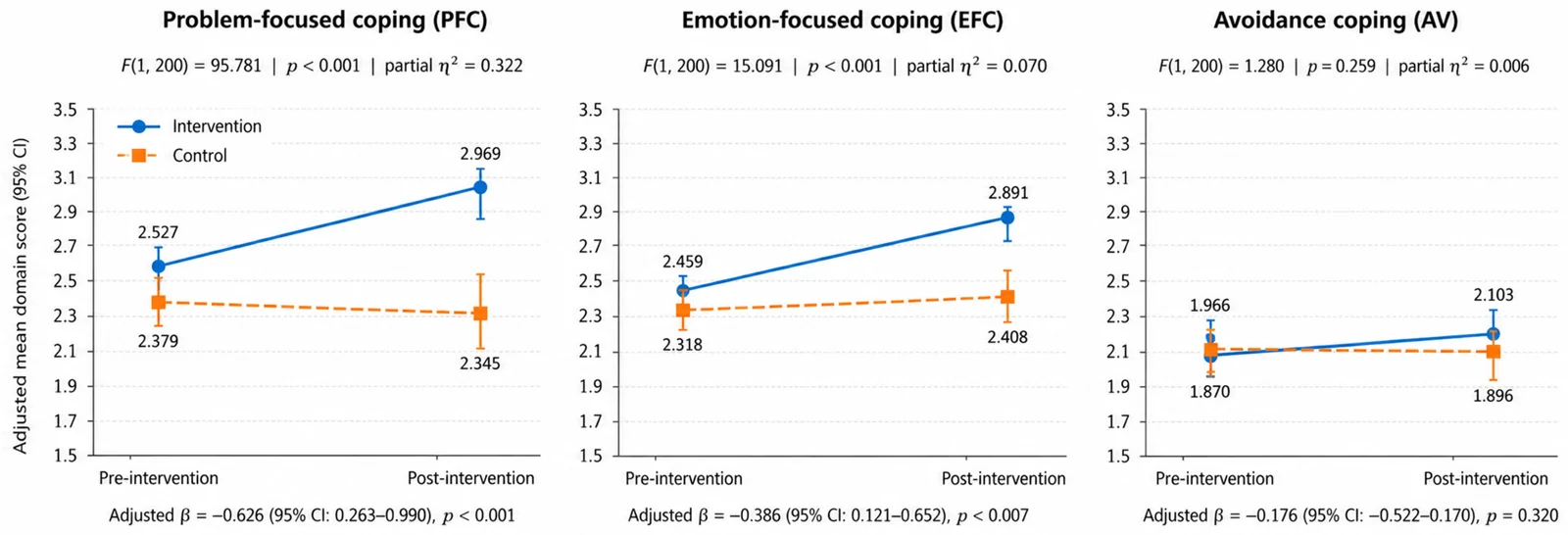
Adjusted longitudinal changes in caregiver coping outcomes by study group. Note: Points represent adjusted estimated marginal means (EMMs), and error bars represent 95% confidence intervals. Adjusted Δ represents the between-group difference in change (group-by-time interaction contrast). Estimates were derived from linear mixed-effects models adjusted for age group and marital status.

Taken together, the primary adjusted longitudinal analyses showed that participation in the web-based digital psychoeducation program was associated with greater increases over time in problem-focused and emotion-focused coping. The corresponding interaction effect sizes were large for problem-focused coping and medium for emotion-focused coping. No statistically significant differential change was identified for avoidance coping. The adjusted longitudinal trajectories for the three coping outcomes are visually presented in Figure 2.

### 3.4. Complementary Unadjusted Analyses

Complementary unadjusted analyses were conducted to characterize within-group changes and between-group differences in change scores across the three coping domains. These analyses were used to support the interpretation of the primary adjusted longitudinal models reported in Section 3.3. Observed pre- and post-intervention scores, within-group changes, and between-group comparisons of change scores are presented in Table 5.

Problem-focused coping increased in the intervention group from 2.67 (SD = 0.55) at baseline to 3.09 (SD = 0.56) at post-intervention, corresponding to a raw mean change of +0.42. The within-group change was statistically significant (Z = −7.345, *p* < 0.001, *r* = 0.73). In the control group, the mean changed from 2.52 (SD = 0.21) to 2.49 (SD = 0.34), with a raw mean change of −0.03; the within-group change was not statistically significant (Z = −1.155, *p* = 0.248, *r* = 0.11). The distribution of change scores differed significantly between groups (U = 1669.00, Z = −8.347, *p* < 0.001, *r* = 0.59).

Emotion-focused coping increased from 2.41 (SD = 0.47) to 2.75 (SD = 0.45) in the intervention group, corresponding to a raw mean change of +0.33. The within-group change was statistically significant (Z = −6.604, *p* < 0.001, *r* = 0.66). In the control group, the mean increased from 2.39 (SD = 0.49) to 2.46 (SD = 0.48), with a raw mean change of +0.07; this change was not statistically significant (Z = −1.365, *p* = 0.172, *r* = 0.14). The distribution of change scores differed significantly between groups (U = 3447.00, Z = −3.990, *p* < 0.001, *r* = 0.28).

Avoidance coping showed little change in either group. The intervention-group mean increased from 2.14 (SD = 0.36) to 2.21 (SD = 0.49), corresponding to a raw mean change of +0.06, and the within-group change was not statistically significant (Z = −1.341, *p* = 0.180, *r* = 0.13). In the control group, the mean changed from 2.18 (SD = 0.35) to 2.17 (SD = 0.34), corresponding to a raw mean change of −0.01, with no significant within-group change (Z = −0.071, *p* = 0.943, *r* = 0.01). The distribution of change scores did not differ significantly between groups (U = 4726.50, Z = −0.902, *p* = 0.367, *r* = 0.06).

### 3.5. Sensitivity Analysis Using Baseline-Adjusted Regression

Baseline-adjusted linear regression models were conducted as sensitivity analyses to assess whether the findings were consistent with those obtained from the primary linear mixed-effects models. For each coping outcome, the post-intervention score was modeled as the dependent variable, with study group, the corresponding baseline coping score, age group, and marital status included as predictors.

For problem-focused coping, study group remained significantly associated with the post-intervention score after adjustment for baseline problem-focused coping, age group, and marital status (B = 0.506, 95% CI: 0.409–0.603, *p* < 0.001). The model accounted for a substantial proportion of variation in post-intervention problem-focused coping (adjusted R^2^ = 0.657).

A significant adjusted association was also observed for emotion-focused coping (B = 0.204, 95% CI: 0.086–0.323, *p* = 0.001; adjusted R^2^ = 0.426). In contrast, the adjusted association between study group and post-intervention avoidance coping was not statistically significant (B = 0.121, 95% CI: −0.016 to 0.257, *p* = 0.082; adjusted R^2^ = 0.044).

The sensitivity analyses were consistent with the primary longitudinal findings, with evidence of between-group differences for problem-focused and emotion-focused coping but not for avoidance coping.

### 3.6. Exploratory Analysis of the Brief COPE Subscales

To examine whether changes in the broader coping domains were reflected in specific coping strategies, exploratory analyses were conducted across all 14 Brief COPE subscales. Pre-to-post change scores were compared between the intervention and control groups using the Mann–Whitney U test, with Holm’s sequential procedure applied across the 14 comparisons to account for multiple testing. Detailed results are provided in Supplementary Table S1.

Before adjustment for multiple comparisons, between-group differences in change scores were observed for active coping, planning, instrumental support, positive reframing, acceptance, religion, humor, substance use, and venting. The largest difference was observed for planning (U = 1878.00, Z = −7.997, *p* < 0.001, *r* = 0.56), followed by instrumental support (U = 2891.00, Z = −5.535, *p* < 0.001, *r* = 0.39). Between-group differences were also found for active coping (U = 3735.50, Z = −3.434, *p* < 0.001, *r* = 0.24) and venting (U = 3711.00, Z = −3.447, *p* < 0.001, *r* = 0.24).

After adjustment for multiple comparisons, the clearest evidence of between-group differences remained for planning, instrumental support, active coping, humor, substance use, and venting. Positive reframing was at the threshold of statistical significance after Holm adjustment (adjusted *p* ≈ 0.050) and was therefore interpreted cautiously. Acceptance and religion, although significant in the unadjusted analyses, did not retain statistical significance after correction. No evidence of between-group differences in change was found for emotional support, self-distraction, denial, behavioral disengagement, or self-blame. Complete results for all 14 subscales, including Mann–Whitney U statistics, standardized Z values, unadjusted and Holm-adjusted *p*-values, and effect sizes, are reported in Supplementary Table S1.

## 4. Discussion

The findings of this study indicate that participation in a web-based digital psychoeducation program was associated with favorable longitudinal changes in selected coping mechanisms among family caregivers of individuals with schizophrenia. Previous psychoeducation studies among caregivers of individuals with schizophrenia have predominantly evaluated broader caregiver outcomes, including caregiver burden, psychological distress, expressed emotion, and illness-related knowledge, while specific changes in coping strategies remain less frequently examined [22,37]. In contrast, the present study specifically examined changes in caregiver coping mechanisms by evaluating three major domains: problem-focused coping, emotion-focused coping, and avoidance coping.

The primary adjusted longitudinal analyses showed greater increases in problem-focused and emotion-focused coping over time in the intervention group relative to the control group, whereas no statistically significant differential change was observed for avoidance coping. These findings are consistent with the possibility that participation in digital psychoeducation may support caregiver adaptation, particularly in coping processes related to problem solving, emotional regulation, and appraisal of caregiving-related challenges [27,38]. However, the present study did not directly evaluate the mechanisms through which these changes occurred.

Caring for individuals with schizophrenia requires continuous adaptation because caregivers frequently encounter complex situations, including unpredictable symptoms, medication management difficulties, relapse concerns, behavioral changes, and uncertainty regarding disease progression [39,40]. These circumstances may become chronic sources of stress for families when caregivers lack sufficient knowledge, caregiving skills, and effective coping strategies [41]. Therefore, strengthening coping mechanisms represents an important component of caregiver interventions because caregivers’ ability to manage stress may influence both the quality of care provided and their own psychological well-being [13,42].

The primary contribution of this study lies in providing evidence of differential longitudinal changes across specific coping domains associated with participation in digital psychoeducation, rather than focusing solely on broader caregiver outcomes. Previous research has shown that psychoeducation provides benefits for families of individuals with schizophrenia by improving illness understanding, reducing caregiver burden, and supporting caregivers in managing the challenges associated with long-term caregiving demands [37]. A recent systematic review and meta-analysis involving 21 studies and 1639 caregivers reported that psychoeducation was associated with improvements in several caregiver outcomes, including caregiver burden, quality of life, and expressed emotion [22]. The study further suggested that the mechanisms underlying psychoeducation may extend beyond knowledge acquisition by influencing coping mechanisms and stress appraisal processes based on Lazarus and Folkman’s transactional model of stress and coping [43].

Previous findings have also demonstrated that psychoeducation can reduce caregiver burden by improving illness understanding and strengthening caregivers’ ability to manage caregiving challenges [21]. However, most previous studies have evaluated psychoeducation outcomes using broader indicators, such as caregiver burden or psychological distress, whereas the modification of specific coping strategies remains relatively underexplored. Therefore, the present study provides additional insight into how digital psychoeducation may influence specific coping responses among caregivers of individuals with schizophrenia.

The intervention was delivered through the Ademind.online web-based platform over a 10-week period consisting of ten structured learning modules. The modules covered schizophrenia knowledge, caregiving skills, stress management, communication strategies, relapse prevention, long-term care planning, resilience, and emotional well-being. The intervention was designed not only to provide information but also to develop caregivers’ practical and psychological resources. Each module included specific learning objectives that guided caregivers to understand schizophrenia, reflect on their caregiving experiences, and apply learned strategies to daily caregiving situations. This structured learning process may help explain the observed changes in coping mechanisms, as caregivers were gradually exposed to new knowledge while developing cognitive and behavioral responses relevant to caregiving challenges.

To ensure that the intervention was implemented as planned, weekly monitoring sheets were used to document completion of each module, allowing the researchers to verify that participants completed the full psychoeducation program and received the intended educational materials. Unlike one-time educational approaches that primarily provide information, repeated exposure to structured learning materials may allow caregivers to integrate new knowledge, reflect on previous caregiving experiences, and develop more adaptive responses to caregiving challenges.

Coping mechanisms in this study were assessed using the Brief COPE Inventory [33]. For the primary domain-level analyses, the 14 original subscales were organized into three broader theory-informed coping domains: problem-focused coping, emotion-focused coping, and an avoidance-oriented coping composite. Problem-focused coping comprised active coping, planning, and instrumental support. Emotion-focused coping comprised emotional support, positive reframing, acceptance, religion, and humor. The avoidance-oriented composite comprised self-distraction, denial, venting, behavioral disengagement, substance use, and self-blame. This organization was used as a theory-informed scoring framework rather than as a psychometrically confirmed higher-order factor structure in the present population.

### 4.1. Effects on Problem-Focused Coping

The most pronounced longitudinal difference between the study groups was observed for problem-focused coping. In the primary adjusted analysis, the group-by-time interaction was statistically significant, *F*(1, 200) = 90.878, *p* < 0.001, with a large effect size (partial ηp^2^ = 0.312). The adjusted estimated marginal mean increased from 2.577 at pre-intervention to 2.998 at post-intervention in the intervention group, whereas it changed from 2.381 to 2.351 in the control group. The adjusted between-group difference in change was 0.450 points (95% CI: 0.357–0.544), indicating a substantially greater increase in problem-focused coping over time among participants in the intervention group.

The complementary unadjusted analyses showed a consistent pattern. The observed mean problem-focused coping score increased from 2.67 (SD = 0.55) to 3.09 (SD = 0.56) in the intervention group, with a significant within-group change (Z = −7.345, *p* < 0.001). In the control group, the mean changed only slightly, from 2.52 (SD = 0.21) to 2.49 (SD = 0.34), and the within-group change was not statistically significant (Z = −1.155, *p* = 0.248). The distributions of pre-to-post change scores also differed significantly between groups (Mann–Whitney U = 1669.00, Z = −8.347, *p* < 0.001). These unadjusted findings support the pattern identified in the primary longitudinal model.

From a caregiving perspective, this finding is relevant because family members caring for individuals with schizophrenia routinely face practical demands such as medication supervision, symptom monitoring, assistance with daily activities, behavioral changes, and recognition of early signs of relapse [44,45]. These responsibilities can become persistent sources of stress when caregivers have limited knowledge or feel uncertain about how to respond to changes in the patient’s condition [15,46]. The intervention addressed several of these demands through modules on basic caregiving, medication management, symptom monitoring, treatment adherence, crisis management, and relapse prevention. Access to structured information and practical guidance may have expanded the resources caregivers perceived as available when responding to caregiving-related problems.

This interpretation is consistent with the Transactional Model of Stress and Coping proposed by Lazarus and Folkman [43], in which coping responses depend partly on how individuals appraise a stressor and the resources they perceive as available to manage it. By providing illness-related knowledge and practical caregiving strategies, the program may have influenced participants’ appraisal of caregiving demands and their perceived capacity to address them. This explanation remains tentative, however, because the non-randomized allocation means that residual or unmeasured differences between the study groups cannot be excluded.

The observed pattern is also consistent with previous evidence indicating that psychoeducation can support caregiver adaptation by improving illness understanding and strengthening resources for managing caregiving demands [22]. In the present study, the large group-by-time interaction suggests that problem-focused coping was the domain in which the clearest differential longitudinal change was observed. Given the quasi-experimental design, this finding is best interpreted as an association with participation in the web-based digital psychoeducation program rather than as proof of a causal intervention effect.

### 4.2. Effects on Emotion-Focused Coping

Emotion-focused coping also showed a significant differential change over time. The primary adjusted analysis identified a significant group-by-time interaction, *F*(1, 200) = 16.907, *p* < 0.001, with a medium effect size (partial ηp^2^ = 0.078). The adjusted estimated marginal mean increased from 2.340 to 2.675 in the intervention group, compared with an increase from 2.336 to 2.407 in the control group. The adjusted between-group difference in change was 0.263 points (95% CI: 0.137–0.390), indicating a greater adjusted longitudinal increase in emotion-focused coping among participants in the intervention group.

The complementary analyses were consistent with the adjusted findings. The observed mean increased from 2.41 (SD = 0.47) to 2.75 (SD = 0.45) in the intervention group, with a significant within-group change (Z = −6.604, *p* < 0.001). In the control group, the mean increased from 2.39 (SD = 0.49) to 2.46 (SD = 0.48), but the within-group change was not statistically significant (Z = −1.365, *p* = 0.172). The distribution of change scores differed significantly between groups (Mann–Whitney U = 3447.00, Z = −3.990, *p* < 0.001).

The emotional demands of caring for a family member with schizophrenia extend beyond the practical tasks of caregiving. Caregivers may experience uncertainty about relapse and the future course of the illness, frustration related to behavioral changes, stigma, and changes in family roles [10,47]. Many of these circumstances cannot be resolved through problem solving alone and may require caregivers to regulate their emotional responses while continuing to provide long-term care.

Several components of the psychoeducation program addressed these needs, including stress management, social support, resilience, and emotional well-being. These components may have provided caregivers with additional resources for recognizing and managing emotional responses arising from caregiving experiences. The finding should not be interpreted as evidence that psychological distress itself was reduced, because distress was not measured as an outcome in this study. Rather, the observed change concerns the coping strategies participants reported using in response to caregiving-related stress.

Within the Transactional Model of Stress and Coping [43], emotion-focused strategies are particularly relevant when aspects of a stressful situation cannot readily be changed. In long-term schizophrenia caregiving, caregivers have limited control over many aspects of illness progression, symptom fluctuation, and treatment response. Strategies that help regulate emotional reactions may therefore complement problem-focused efforts directed toward caregiving situations that are more amenable to action. Previous research has similarly suggested that psychoeducational interventions may support psychological adjustment through improved illness understanding, stress-management skills, and emotional resources [21,22,23].

Digital delivery may also have allowed participants to engage with educational materials according to their individual circumstances and revisit content when needed. Repeated exposure over the 10-week program may have provided opportunities to reflect on caregiving experiences and gradually incorporate the material into their responses to everyday stressors. This interpretation remains provisional, as the study design does not allow the observed between-group difference to be attributed exclusively to the intervention.

The magnitude of the adjusted effects provides additional context for interpreting the statistical findings. The group-by-time interaction for problem-focused coping was large (partial ηp^2^ = 0.312), whereas the corresponding effect for emotion-focused coping was moderate (partial ηp^2^ = 0.078). The adjusted difference in longitudinal change was 0.450 points for problem-focused coping and 0.263 points for emotion-focused coping on the original 1–4 response metric. These estimates indicate that the findings were accompanied by measurable differences in change between the study groups rather than statistical significance alone.

Established thresholds for clinically meaningful change are not available for the theory-informed Brief COPE composite scores used in this study. The observed differences therefore cannot be classified as having reached a predefined clinically important threshold. Their practical relevance is better considered in relation to the magnitude and precision of the adjusted estimates, the corresponding confidence intervals, and the consistency of the findings across the primary and complementary analyses. This distinction is important because a statistically significant difference does not, by itself, establish clinical importance.

### 4.3. Interpretation of Avoidance Coping Changes

The pattern for avoidance coping differed from those observed for problem-focused and emotion-focused coping. In the primary adjusted analysis, the group-by-time interaction was not statistically significant, *F*(1, 200) = 1.283, *p* = 0.259, and the corresponding effect size was small (partial ηp^2^ = 0.006). The adjusted estimated marginal mean changed from 2.050 to 2.113 in the intervention group and from 2.078 to 2.066 in the control group. The adjusted between-group difference in change was 0.074 points (95% CI: −0.055 to 0.204). The confidence interval included zero, providing insufficient evidence that longitudinal changes in avoidance coping differed between the study groups.

The complementary unadjusted analyses led to the same interpretation. The observed mean in the intervention group changed from 2.14 (SD = 0.36) to 2.21 (SD = 0.49), but the within-group change was not statistically significant (Z = −1.341, *p* = 0.180). In the control group, the mean changed from 2.18 (SD = 0.35) to 2.17 (SD = 0.34), also without a significant within-group change (Z = −0.071, *p* = 0.943). The between-group comparison of change scores was likewise not statistically significant (Mann–Whitney U = 4726.50, Z = −0.902, *p* = 0.367).

These findings reinforce an important distinction between change within a study group and evidence of an intervention-associated difference. A pre-to-post change observed within one group does not establish an intervention effect unless the longitudinal pattern differs from that of the comparison group. In this study, neither the primary group-by-time interaction nor the complementary comparison of change scores provided evidence of a differential change in the broader avoidance coping domain.

Interpretation of this domain also requires consideration of its heterogeneous composition. The avoidance-oriented composite comprised self-distraction, denial, venting, behavioral disengagement, substance use, and self-blame, based on the theory-informed organization of the Brief COPE subscales adopted in this study [35]. These strategies may differ in their psychological function and in how they are used in response to caregiving-related stress [10,13,42]. The poor internal consistency observed for the avoidance-oriented composite in the present sample (Cronbach’s α = 0.245) further indicates limited measurement reliability and suggests that this broader domain should not be interpreted as a homogeneous coping construct.

The supplementary analysis of the 14 individual Brief COPE subscales provides a more granular view of this heterogeneity. Strategy-specific differences were observed for some subscales but not for others, and several findings did not remain statistically significant after Holm adjustment for multiple comparisons. Thus, changes at the level of individual coping strategies should not be generalized to the broader avoidance-oriented composite. This subscale-level pattern also supports cautious interpretation of the three-domain framework, particularly for a composite with limited internal consistency.

The content of the intervention may provide another explanation for the absence of a differential change in avoidance coping. The program primarily addressed illness-related knowledge, practical caregiving skills, stress management, communication, social support, relapse prevention, resilience, and emotional well-being. It was not specifically designed to reduce avoidance-related behaviors. This difference in intervention focus may partly explain why clearer longitudinal changes were observed for problem-focused and emotion-focused coping than for the broader avoidance-oriented domain.

Future studies could examine individual Brief COPE strategies longitudinally rather than relying solely on higher-order composite scores. More targeted coping-skills interventions could also be considered when avoidance-related responses are an explicit intervention target. Longer follow-up periods may help determine whether changes in these strategies emerge after caregivers have had more time to apply newly acquired skills in everyday caregiving situations.

### 4.4. Scope of the Study Outcomes

The present study was designed to examine caregiver coping as the principal outcome of the web-based digital psychoeducation program. Although several intervention modules addressed stress management, social support, resilience, emotional well-being, communication, and long-term care planning, these components were included as part of the psychoeducational content intended to support caregivers in managing caregiving demands rather than as separate outcomes of the present analysis. Accordingly, caregiver burden, psychological distress, quality of life, resilience, perceived stress, social support, and caregiving competence were not assessed as study outcomes.

This outcome focus should be considered when interpreting the findings. Improvements in problem-focused and emotion-focused coping cannot be assumed to represent corresponding reductions in caregiver burden or psychological distress, nor can they be interpreted as evidence of improved quality of life, resilience, social support, or caregiving competence. These constructs are related but conceptually distinct aspects of the caregiving experience and require direct assessment. Future evaluations of web-based digital psychoeducation should therefore incorporate a broader set of caregiver outcomes, including validated multidimensional measures of caregiver burden and care-related quality of life alongside coping assessments, to determine whether changes in coping are accompanied by meaningful changes in caregiver well-being and functioning.

### 4.5. Strengths and Limitations

This study has several strengths. First, it contributes to the growing evidence on the potential role of web-based digital psychoeducation in supporting coping mechanisms among family caregivers of individuals with schizophrenia. While previous studies have primarily examined broader caregiver outcomes, such as caregiver burden, psychological distress, and illness-related knowledge, the present study specifically assessed changes across three coping domains: problem-focused coping, emotion-focused coping, and avoidance coping. This broader assessment provides a more detailed perspective on how caregivers may respond to the psychological demands of long-term caregiving.

A further strength was the development of a structured web-based intervention specifically for family caregivers of individuals with schizophrenia. The program consisted of ten sequential modules covering schizophrenia knowledge, caregiving strategies, stress management, communication skills, social support, relapse prevention, long-term care planning, resilience, and emotional well-being. The intervention was developed using the ADDIE framework and was evaluated by multidisciplinary experts from psychiatry, clinical psychology, mental health nursing, and academic nursing. This process helped ensure that the content was relevant to the caregiving context and informed by both clinical and educational perspectives.

Another strength was the inclusion of both intervention and control groups, which allowed changes in coping mechanisms to be examined over the same study period. The use of the Brief COPE Inventory enabled examination of multiple coping strategies and theory-informed coping domains rather than relying on a single global measure of caregiver adaptation. In addition, the primary longitudinal analysis used linear mixed-effects models to evaluate group-by-time interactions while adjusting for age group and marital status. Baseline-adjusted linear regression models were also conducted as sensitivity analyses, providing an alternative adjustment strategy to assess the robustness of the primary findings. Complementary unadjusted analyses were retained to describe within-group changes and between-group differences in change scores.

Several limitations should be considered when interpreting the findings. First, participants were not randomly allocated to the intervention and control groups. Baseline differences were observed between the intervention and control groups, particularly in age, marital status, and problem-focused coping. These differences limit the ability to attribute the observed changes solely to participation in the intervention. Although the primary longitudinal analyses adjusted for age group and marital status, and sensitivity analyses additionally accounted for baseline coping scores, statistical adjustment cannot fully eliminate residual confounding from unmeasured or inadequately measured factors inherent in a non-randomized design. In addition, seven initially recruited intervention participants did not complete the study protocol and were not included in the final analytic sample; additional eligible caregivers were recruited to maintain the established intervention-group quota. Although the final analytic dataset contained complete pre- and post-intervention observations, exclusion of participants without post-intervention data may have introduced attrition-related selection bias. Therefore, the observed between-group differences in longitudinal change should be interpreted as adjusted associations rather than definitive causal effects of the intervention.

Second, the study was conducted at a single mental health hospital in Bengkulu, Indonesia. The characteristics of caregivers, healthcare services, and access to digital technology may differ across geographical and cultural settings. Therefore, cultural, social, healthcare-system, and contextual differences should be considered when extrapolating these findings to caregivers in other populations and settings. Multicenter studies involving caregivers from different regions and healthcare settings would be useful to determine whether similar patterns of coping change can be observed elsewhere.

Third, coping mechanisms were assessed using the Brief COPE Inventory, which is a self-report instrument. Participants’ responses may have been influenced by social desirability, recall, personal interpretation, or their emotional state at the time of assessment [48]. In addition, the internal consistency of the broader coping composites varied in the present sample. Although problem-focused coping showed acceptable internal consistency (Cronbach’s α = 0.716), internal consistency was lower for emotion-focused coping (α = 0.573) and particularly poor for the avoidance-oriented coping composite (α = 0.245). The low reliability of these broader composites, especially avoidance coping, may have reduced measurement precision and should be considered when interpreting the corresponding domain-level findings. To reduce reliance on these composite scores, all 14 original Brief COPE subscales were additionally examined in supplementary analyses. Furthermore, the three-domain composite structure has not been specifically validated among Indonesian family caregivers of people with schizophrenia and should therefore be regarded as a theory-informed scoring framework rather than a confirmed latent structure in this population.

Another limitation concerns the scope of the outcomes assessed. The study focused on coping mechanisms and did not include direct measures of caregiver burden, psychological distress, quality of life, resilience, perceived stress, social support, or caregiving competence. Although several of these domains were addressed within the psychoeducation program, their inclusion as intervention content does not establish that they improved following participation. It therefore remains unclear whether the observed changes in coping were accompanied by reductions in caregiver burden or distress, improvements in quality of life, or changes in other dimensions of caregiver well-being. In addition, clinical outcomes of the individuals with schizophrenia were not assessed; therefore, it cannot be determined whether changes in caregivers’ coping were accompanied by changes in patients’ symptoms, relapse, treatment adherence, or functioning. Future studies should evaluate these outcomes alongside coping measures to provide a more comprehensive assessment of the potential benefits of digital psychoeducation for family caregivers.

Another limitation is the absence of medium- or long-term follow-up assessments. Coping outcomes were assessed immediately before and after the 10-week intervention, and the study therefore cannot determine whether the observed differences in problem-focused and emotion-focused coping were maintained beyond the intervention period. Future studies should include additional follow-up assessments to examine the durability of changes in caregiver coping over time.

Attrition should also be considered when interpreting the findings. Seven participants initially enrolled in the intervention group did not complete the study protocol and did not contribute post-intervention data. The final analyses were therefore based on participants with complete pre- and post-intervention assessments, and no imputation of missing post-intervention outcomes was performed. Although additional eligible caregivers were recruited until the intervention-group recruitment quota was reached, the exclusion of non-completers from the final analyses may have introduced attrition-related selection bias if those who discontinued differed systematically from participants who completed the intervention.

The digital mode of intervention delivery introduces an additional consideration regarding participant selection and generalizability. Eligibility required access to an internet-connected digital device, which may have favored caregivers who had greater access to digital resources or were more comfortable using web-based services. Caregivers experiencing limited internet availability, financial constraints related to connectivity, restricted access to digital devices, or difficulty using digital technologies may therefore have been less likely to participate. This potential digital divide is particularly relevant when considering the applicability of web-based psychoeducation in resource-constrained settings, where access to digital infrastructure may not be evenly distributed.

The study did not systematically collect information on the type of device used to access the intervention, the quality or stability of participants’ internet connections, previous experience with digital technologies, or digital literacy using a standardized measure. Consequently, differences in intervention engagement according to these characteristics could not be examined. The relationship of age and educational level with intervention adherence was also not formally evaluated. These limitations restrict conclusions regarding whether the program is equally accessible and feasible across caregivers with different levels of digital readiness. Accordingly, the high completion observed among participants who remained in the intervention should not be interpreted as evidence that the program would be equally feasible for caregivers with limited digital access or lower digital literacy. Future studies should systematically assess device type, internet accessibility and stability, previous experience with digital technologies, and digital literacy using validated measures, and examine whether these characteristics, together with age and educational level, are associated with intervention uptake, adherence, and outcomes.

## 5. Conclusions

Participation in the 10-week web-based digital psychoeducation program was associated with greater adjusted longitudinal improvements in problem-focused and emotion-focused coping among family caregivers of people with schizophrenia. The adjusted between-group difference in change was 0.450 points (95% CI: 0.357–0.544) for problem-focused coping and 0.263 points (95% CI: 0.137–0.390) for emotion-focused coping. In contrast, no statistically significant differential change was observed for avoidance coping (adjusted difference in change = 0.074 points, 95% CI: −0.055 to 0.204).

These findings suggest that participation in web-based digital psychoeducation may be associated with favorable changes in selected coping domains among family caregivers of people with schizophrenia. However, given the non-randomized quasi-experimental design, observed baseline differences between groups, and the possibility of residual confounding, the findings should not be interpreted as definitive evidence of causal efficacy. Further randomized or multicenter studies with longer follow-up periods and broader caregiver and patient outcomes are needed to confirm these findings and evaluate their longer-term practical and clinical relevance.

## 6. Implications for Practice and Future Research

From a clinical perspective, the findings of this study suggest that web-based digital psychoeducation may serve as a complementary approach within mental health services to extend caregiver support beyond conventional hospital-based education. Family caregivers of individuals with schizophrenia require continuous education and psychological support because caregiving responsibilities are often prolonged and associated with fluctuating clinical conditions. However, structured caregiver support programs remain limited in many healthcare settings, particularly in regions where mental health resources, healthcare personnel, and access to face-to-face interventions are constrained.

The findings of the present study indicate that participation in the web-based psychoeducation program was associated with greater improvements in problem-focused and emotion-focused coping compared with the control group. Given the non-randomized design and the presence of baseline differences between groups, these findings should be interpreted as an association rather than definitive evidence of a causal intervention effect. Nevertheless, the results suggest that structured digital psychoeducation may have practical value as part of a broader caregiver support strategy, particularly when conventional face-to-face services are difficult to access.

The integration of structured digital caregiver programs into routine schizophrenia care may provide healthcare professionals with an additional strategy to deliver continuous psychoeducational support, improve illness-related knowledge, and support the development of adaptive coping skills among caregivers. The web-based format allows caregivers to access educational materials flexibly according to their individual circumstances, potentially reducing barriers related to geographical distance, transportation difficulties, time constraints, and competing caregiving responsibilities. Therefore, digital psychoeducation should be considered a complementary intervention that can strengthen existing family-centered mental health services rather than replace direct clinical interactions.

For mental health nurses and other healthcare professionals, these findings highlight the potential role of digital platforms in extending caregiver support beyond traditional clinical encounters. Digital psychoeducation programs may facilitate continuous education, reinforcement of caregiving skills, and opportunities for caregivers to revisit information when facing challenging situations. However, successful implementation is likely to depend on caregiver digital literacy, access to reliable internet and digital devices, engagement with the learning materials, and appropriate professional support. These factors should be considered when integrating web-based caregiver interventions into routine mental health services.

Future implementation studies should examine how digital caregiver support programs can be integrated into routine schizophrenia management pathways, including their feasibility, acceptability, adherence, cost-effectiveness, scalability, and sustainability. Further research should also assess whether improvements in coping mechanisms are accompanied by changes in caregiver burden, psychological well-being, quality of life, and patient-related outcomes. Such studies would help determine whether changes in coping mechanisms observed in the present study translate into broader benefits for caregivers and their families.

Future research should also consider multicenter randomized controlled trials with adequate control of baseline characteristics and longer follow-up periods to provide stronger evidence regarding the causal effects and sustainability of digital psychoeducation. Because the present study identified baseline differences between the intervention and control groups, future studies should incorporate random allocation where feasible or apply robust longitudinal and baseline-adjusted analytical approaches when randomization is not possible. In addition, future interventions may incorporate more targeted psychological approaches, such as cognitive behavioral strategies, acceptance-based interventions, or structured coping-skills training, particularly to address avoidance-related coping patterns that were not significantly different between groups in the present study.

Further investigation is also warranted to determine whether web-based digital psychoeducation can be effectively integrated with existing mental health nursing and community-based services in Indonesia. Research examining implementation across different healthcare settings and caregiver populations may provide important evidence regarding the accessibility, acceptability, and scalability of digital caregiver support. Such evidence could inform the development of sustainable, family-centered digital mental health services for caregivers of individuals with schizophrenia.

## Figures and Tables

**Table 1:** Structure and Content of the Web-Based Digital Psychoeducation Modules.

Module and Topic	Key Content	Expected Learning Outcome
Module 1. Introduction to Schizophrenia	Definition, diagnostic characteristics, common symptoms, possible causes, and the importance of early recognition.	Caregivers understand the basic characteristics, symptoms, and contributing factors of schizophrenia and are better able to respond to caregiving challenges.
Module 2. Impact of Schizophrenia on the Family	Emotional and social impact of schizophrenia on caregivers and family members, including grief, frustration, isolation, family dynamics, communication, and support.	Caregivers recognize the emotional and social impact of schizophrenia and become more aware of their psychological responses to caregiving responsibilities.
Module 3. Basic Care for People with Schizophrenia	Daily caregiving practices, medication management and supervision, symptom monitoring, treatment adherence, establishing routines, and medication side effects.	Caregivers acquire practical knowledge and skills for daily care, medication supervision, symptom monitoring, and treatment adherence.
Module 4. Stress Management for Caregivers	Stress reduction techniques, mindfulness, deep-breathing exercises, physical activity, self-care, and seeking support.	Caregivers learn practical strategies to manage stress and maintain their psychological well-being while providing long-term care.
Module 5. Importance of Social Support	Family and peer support, community resources, support groups, and available mental health services.	Caregivers identify available sources of social support and develop strategies to strengthen their support networks.
Module 6. Effective Communication with People with Schizophrenia	Active listening, validation, non-verbal communication, trust-building, and strategies for understanding patients’ needs.	Caregivers develop effective communication skills that promote supportive interactions and improve understanding of patients’ needs.
Module 7. Managing Crisis and Relapse	Early warning signs of relapse, crisis recognition, crisis management planning, and appropriate responses during emergencies.	Caregivers recognize early signs of relapse and develop appropriate strategies for responding to crises while maintaining patient and caregiver safety.
Module 8. Long-Term Care Planning	Patient needs and preferences, flexible care planning, recovery support, daily functioning, and anticipated changes in the patient’s condition.	Caregivers develop realistic and flexible long-term care plans that address patients’ needs, preferences, recovery, and daily functioning.
Module 9. Building Resilience and Emotional Well-Being	Resilience-building strategies, goal setting, positive thinking, social connectedness, and prioritizing caregivers’ emotional health.	Caregivers strengthen resilience and develop strategies to maintain emotional well-being during long-term caregiving.
Module 10. Evaluation and Reflection	Review of learning materials, reflection on caregiving experiences, assessment of preparedness, and planning for future caregiving.	Caregivers consolidate their learning, reflect on their caregiving experiences, and develop plans for applying the knowledge and skills gained.

**Table 2:** Baseline Demographic Characteristics of Family Caregivers of People with Schizophrenia.

Variable	Category	Total N (%)	Intervention N (%)	Control N (%)	χ^2^	*p*-Value
Sex	Male	95 (47.0)	53 (52.5)	42 (41.6)	2.405	0.121
	Female	107 (53.0)	48 (47.5)	59 (58.4)		
Age group	18–25 years	31 (15.3)	25 (24.8)	6 (5.9)	23.214	<0.001
	26–35 years	63 (31.2)	36 (35.6)	27 (26.7)		
	36–45 years	30 (14.9)	14 (13.9)	16 (15.8)		
	46–55 years	44 (21.8)	12 (11.9)	32 (31.7)		
	56–65 years	34 (16.8)	14 (13.9)	20 (19.8)		
Marital status	Not married	48 (23.8)	32 (31.7)	16 (15.8)	6.996	0.008
	Married	154 (76.2)	69 (68.3)	85 (84.2)		
Educational level	No formal education	6 (3.0)	2 (2.0)	4 (4.0)	10.844	0.093
	Primary school	17 (8.4)	7 (6.9)	10 (9.9)		
	Junior high school	30 (14.9)	15 (14.9)	15 (14.9)		
	Senior high school/Vocational school	84 (41.6)	49 (48.5)	35 (34.7)		
	Diploma	13 (6.4)	2 (2.0)	11 (10.9)		
	Bachelor’s degree (S1)	48 (23.8)	25 (24.8)	23 (22.8)		
	Postgraduate degree (S2/S3)	4 (2.0)	1 (1.0)	3 (3.0)		
Occupation	Unemployed	38 (18.8)	18 (17.8)	20 (19.8)	0.641	0.986
	Student	13 (6.4)	7 (6.9)	6 (5.9)		
	Farmer	43 (21.3)	21 (20.8)	22 (21.8)		
	Private-sector employee	46 (22.8)	25 (24.8)	21 (20.8)		
	Civil servant	15 (7.4)	7 (6.9)	8 (7.9)		
	Entrepreneur	47 (23.3)	23 (22.8)	24 (23.8)		
Monthly family income	<IDR 1,000,000	48 (23.8)	22 (21.8)	26 (25.7)	3.580	0.466
	IDR 1,000,000–2,999,999	93 (46.0)	53 (52.5)	40 (39.6)		
	IDR 3,000,000–4,999,999	40 (19.8)	17 (16.8)	23 (22.8)		
	IDR 5,000,000–9,999,999	17 (8.4)	7 (6.9)	10 (9.9)		
	≥IDR 10,000,000	4 (2.0)	2 (2.0)	2 (2.0)		

Note: Values are presented as *n* (%). Percentages for the total sample are based on N = 202, whereas percentages for the intervention and control groups are based on *n* = 101 each. Pearson’s chi-square test was used for categorical comparisons. For marital status, not married combines unmarried, divorced, and widowed participants because of the very small frequencies in the divorced and widowed categories. Statistical significance was set at *p* < 0.05.

**Table 3:** Baseline Coping Outcomes in the Intervention and Control Groups.

Coping Outcome	Intervention Mean (SD)	Median (IQR)	Control Mean (SD)	Median (IQR)	Mann–Whitney U	*p*-Value
Problem-focused coping	2.67 (0.55)	2.67 (0.67)	2.52 (0.21)	2.50 (0.33)	3493.50	<0.001
Emotion-focused coping	2.41 (0.47)	2.50 (0.70)	2.39 (0.49)	2.40 (0.75)	4853.50	0.551
Avoidance coping	2.14 (0.36)	2.17 (0.58)	2.18 (0.35)	2.17 (0.50)	4859.00	0.560

Note: Values are presented as mean (SD) and median (IQR). Between-group comparisons were performed using the Mann–Whitney U test because the coping score distributions did not consistently satisfy the assumptions required for parametric comparisons. All tests were two-tailed, with *p* < 0.05 considered statistically significant. Primary inference regarding differential change over time was based on the adjusted Group × Time interaction from the linear mixed-effects models reported in Section 3.3.

**Table 4:** Adjusted Longitudinal Changes in Coping Outcomes Based on Linear Mixed-Effects Models.

Coping Outcome	Intervention Pre EMM (95% CI)	Intervention Post EMM (95% CI)	Control Pre EMM (95% CI)	Control Post EMM (95% CI)	Adjusted Between-Group Difference in Change (95% CI)	*F* (1, 200)	*p*-Value	Partial ηp^2^
Problem-focused coping	2.577 (2.398–2.756)	2.998 (2.815–3.180)	2.381 (2.193–2.568)	2.351 (2.160–2.541)	0.450 (0.357–0.544)	90.878	<0.001	0.312
Emotion-focused coping	2.340 (2.152–2.529)	2.675 (2.488–2.862)	2.336 (2.139–2.533)	2.407 (2.211–2.603)	0.263 (0.137–0.390)	16.907	<0.001	0.078
Avoidance coping	2.050 (1.907–2.193)	2.113 (1.964–2.262)	2.078 (1.928–2.227)	2.066 (1.911–2.221)	0.074 (−0.055–0.204)	1.283	0.259	0.006

Note: EMM = estimated marginal mean; CI = confidence interval; ηp^2^ = partial eta squared. The adjusted between-group difference in change represents the Group × Time interaction contrast. All tests were two-tailed, with *p* < 0.05 considered statistically significant.

**Table 5:** Complementary Unadjusted Analyses of Pre- to Post-Intervention Changes in Coping Outcomes.

Coping Outcome	Study Group	Pre, Mean (SD)	Post, Mean (SD)	Raw Mean Change	Within-Group Z	*p*	*r*	Between-Group Change U	Z	*p*	*r*
Problem-focused coping	Intervention	2.67 (0.55)	3.09 (0.56)	+0.42	−7.345	<0.001	0.73	1669.00	−8.347	<0.001	0.59
	Control	2.52 (0.21)	2.49 (0.34)	−0.03	−1.155	0.248	0.11				
Emotion-focused coping	Intervention	2.41 (0.47)	2.75 (0.45)	+0.33	−6.604	<0.001	0.66	3447.00	−3.990	<0.001	0.28
	Control	2.39 (0.49)	2.46 (0.48)	+0.07	−1.365	0.172	0.14				
Avoidance coping	Intervention	2.14 (0.36)	2.21 (0.49)	+0.06	−1.341	0.180	0.13	4726.50	−0.902	0.367	0.06
	Control	2.18 (0.35)	2.17 (0.34)	−0.01	−0.071	0.943	0.01				

Note: Values are unadjusted observed means and standard deviations. Raw mean change was calculated as the post-intervention score minus the pre-intervention score. Within-group changes were assessed using the Wilcoxon signed-rank test, and between-group differences in change scores were assessed using the Mann–Whitney U test. Effect size r was calculated as |Z|/√N, using N = 101 for within-group comparisons and N = 202 for between-group comparisons. All tests were two-tailed, with *p* < 0.05 considered statistically significant. Primary inference regarding differential change over time was based on the adjusted Group × Time interaction from the linear mixed-effects models reported in Section 3.3.

## Data Availability

The datasets used and/or analyzed during the current study are available from the corresponding author upon reasonable request. Public sharing of the dataset is restricted due to the sensitive nature of the collected information, including health-related data from family caregivers of individuals with schizophrenia. Any request for data access will be evaluated according to ethical requirements and applicable privacy regulations.

## Supplementary Materials

The following supporting information can be downloaded at https://www.mdpi.com/article/10.3390/healthcare14182888/s1. Supplementary Table S1: Between-Group Comparisons of Pre-to-Post Change Scores Across the 14 Brief COPE Subscales.

## Abbreviations

The following abbreviations are used in this manuscript:

ADDIE	Analysis, Design, Development, Implementation, and Evaluation
Brief COPE	Brief Coping Orientation to Problems Experienced Inventory
CI	Confidence Interval
CVI	Content Validity Index
EMM	Estimated Marginal Mean
IQR	Interquartile Range
LMM	Linear Mixed-Effects Model
S-CVI/Ave	Scale-level Content Validity Index/Average
SD	Standard Deviation
ηp^2^	Partial Eta Squared
