# Supplementary material for: Web-Based Psychoeducation Program for Improving Coping Mechanisms Among Family Caregivers of People with Schizophrenia: A Quasi-Experimental Study
Source: Healthcare (Basel). 2026 Sep 8;14(18):2888. doi: 10.3390/healthcare14182888 (PMC13606837; doi:10.3390/healthcare14182888)
Supplement: Supplementary file 1 [file healthcare-14-02888-s001.zip › healthcare-4533497-supplementary.pdf]

Supplementary Table S1. Between-Group Comparisons of Pre-to-Post Change Scores Across the 14 Brief COPE Subscales

Brief COPE subscale	Mann–Whitney U	Z	Unadjusted *p*-value	Holm-adjusted *p*-value	Effect size (*r*)
Active coping	3735.50	−3.434	<0.001	**0.007**	0.24
Planning	1878.00	−7.997	<0.001	**<0.001**	0.56
Instrumental support	2891.00	−5.535	<0.001	**<0.001**	0.39
Emotional support	4860.50	−0.598	0.550	1.000	0.04
Positive reframing	3996.00	−2.737	0.006	**0.050†**	0.19
Acceptance	4047.50	−2.598	0.009	0.066	0.18
Religion	4237.00	−2.123	0.034	0.203	0.15
Humor	3887.00	−2.948	0.003	**0.032**	0.21
Self-distraction	4337.00	−1.853	0.064	0.319	0.13
Denial	4690.00	−0.999	0.318	1.000	0.07
Substance use	3957.50	−2.780	0.005	**0.049**	0.20
Behavioral disengagement	5027.50	−0.178	0.859	1.000	0.01
Venting	3711.00	−3.447	<0.001	**0.007**	0.24
Self-blame	5011.50	−0.216	0.829	1.000	0.02

Note: *Between-group comparisons were based on pre-to-post change scores and were performed using the Mann–Whitney U test. Effect size r was calculated as* $|Z|/\sqrt{N}$*, with N = 202. Holm's sequential procedure was applied across the 14 subscale comparisons to control the family-wise error rate. For the Holm adjustment, two-sided probabilities corresponding to the standardized Z statistics were used because probabilities below 0.001 were displayed as 0.000 in the SPSS output. Adjusted p-values greater than 1.000 were reported as 1.000. Bold values indicate Holm-adjusted* $p \leq 0.05$*. †The Holm-adjusted p-value for positive reframing was approximately 0.0496 based on the reported Z statistic and is shown as 0.050 after rounding; this finding should therefore be interpreted cautiously.*
Abbreviations: U, Mann–Whitney U statistic; Z, standardized test statistic; *r*, effect size.
